# Bayesian Device-Free Localization and Tracking in a Binary RF Sensor Network

**DOI:** 10.3390/s17050969

**Published:** 2017-04-27

**Authors:** Zhenghuan Wang, Heng Liu, Shengxin Xu, Xiangyuan Bu, Jianping An

**Affiliations:** School of Information and Electronics, Beijing Institute of Technology, Beijing 100081, China; wangzhenghuan@bit.edu.cn (Z.W.); xusx@bit.edu.cn (S.X.); bxy@bit.edu.cn (X.B.); an@bit.edu.cn (J.A.)

**Keywords:** device-free localization (DFL), RSS, energy efficiency, maximum likelihood, particle filter

## Abstract

Received-signal-strength-based (RSS-based) device-free localization (DFL) is a promising technique since it is able to localize the person without attaching any electronic device. This technology requires measuring the RSS of all links in the network constituted by several radio frequency (RF) sensors. It is an energy-intensive task, especially when the RF sensors work in traditional work mode, in which the sensors directly send raw RSS measurements of all links to a base station (BS). The traditional work mode is unfavorable for the power constrained RF sensors because the amount of data delivery increases dramatically as the number of sensors grows. In this paper, we propose a binary work mode in which RF sensors send the link states instead of raw RSS measurements to the BS, which remarkably reduces the amount of data delivery. Moreover, we develop two localization methods for the binary work mode which corresponds to stationary and moving target, respectively. The first localization method is formulated based on grid-based maximum likelihood (GML), which is able to achieve global optimum with low online computational complexity. The second localization method, however, uses particle filter (PF) to track the target when constant snapshots of link stats are available. Real experiments in two different kinds of environments were conducted to evaluate the proposed methods. Experimental results show that the localization and tracking performance under the binary work mode is comparable to the those in traditional work mode while the energy efficiency improves considerably.

## 1. Introduction

When a target (person) enters a monitored region surrounded by a network of radio frequency (RF) sensors, the target will influence the of radio signals emitted by sensors, e.g., shadow and reflect the radio signals [1]. Device-free Localization (DFL) methods exploit this characteristic to localize the target. Compared to device-based localization methods, DFL does not require the target to wear any electronic devices, which is very promising in some emergency scenarios, for instance, search and rescue. Moreover, since the radio signals can penetrate walls and other non-metallic structures, DFL is able to find the targets behind walls [2,3,4]. Radar technology has been employed as a DFL approach for a long time. However, radar relies on the detection of the weak echoes reflected by the target and thus high bandwidth and large power consumption are essential [1]. In the past few years, received-signal-strength-based (RSS-based) DFL methods have gained a lot of attention because RSS measurements are available in most commercial off-the-shelf (COTS) wireless products, which can greatly reduce the cost of DFL systems. So far, RSS-based DFL methods have been successfully applied to environment monitoring [1,2,5,6,7], personnel tracking [2,8,9] and health-care [10,11].

In DFL a number of RF sensors are usually placed in the monitored region and the role of the RF sensors is to measure the RSS of the links comprised of the sensors. In outdoors the RF sensors are usually powered by batteries with limited battery power and in most literature the RF sensors operate in the traditional work mode [1], in which the RF sensors directly transmit the raw RSS measurements of all links to the base station (BS). As a consequence, the energy problem of DFL in traditional work mode arises especially when a large number of sensors are deployed to cover a large area. Specifically, we know that the total number of links in the network comprised of *K* sensors is approximately proportional to $K^{2}$. Thus, the energy consumed by sending measurements grows rapidly as the increase of the number of sensors, which is disadvantageous for batteries powered sensors. In this paper, we propose a new work mode in which the RF sensors only output two states of a link: obstructed and unobstructed. In fact, a target only occupies a little space compared to the entire monitored region, meaning that most links are not affected. Therefore, it can be proved that the amount of data transmission approximately increases linearly rather than quadratically with the number of sensors, which is beneficial to the batteries powered RF sensors. The new work mode can be easily implemented by adding a little local processing to the RF sensors in the traditional work mode.

Moreover, to localize the device-free target when the sensors work in the binary mode, it is necessary to develop new localization methods since previous localization methods [1,8,12,13,14] can only work in the traditional work mode. To address this issue, we reformulate the localization problem under the Bayesian framework for the binary work mode. We consider two scenarios, according to the motion of the target. In the first scenario, the target is stationary and Bayesian estimation degrades to maximum likelihood (ML) estimation [15] if we have no prior knowledge of the position of the target. Since the ML estimation involves optimizing a highly nonlinear objective function, we propose a grid-based ML (GML) method which overcomes local convergence frequently encountered in the iterative optimization methods. We also prove that the computational complexity of GML could be remarkably reduced since the most computation task can be finished offline.

In the second scenario, however, the target is moving within the monitored region and link states are constantly updated, making it possible to track the target. The classical Kalman filter, which is a type of Bayesian filter for the linear/Gaussian case, is not suitable anymore due to the nonlinearity of the link state model. Fortunately, in recent years particle filter (PF) has been widely employed to deal with nonlinear filtering [8,9,12,16,17,18,19,20,21]. In this paper, we perform target tracking in the binary work mode by employing PF which proves to be able to accurately estimate the position of the target.

Finally, we conducted real experiments to verify the effectiveness of the proposed method. We used 20 RF sensors which surrounded a monitored region of 9.5 m × 9.5 m. The aforementioned two scenarios were both taken into consideration in the experiments. The experimental results show that the proposed method can achieve noticeable power reduction as well as comparable localization performance with the localization method in the traditional mode.

The rest of the paper is organized as follows. Section 2 reviews the work related to DFL. Section 3 describes the system model of DFL and introduces the new work mode of sensors. Section 4 compares the energy consumption of the binary work mode with that of the traditional mode. Section 5 presents the GML estimation method for the stationary target scenario. Section 6 provides target tracking framework of DFL using the particle filter. Section 7 presents the experimental results and Section 8 concludes the paper.

## 2. Related Work

Since DFL technology is promising in a great number of scenarios, in the past few years lots of work have been conducted to improve the performance of DFL. In this section, we will give a brief literature review of previous work on DFL technology.

The existing DFL methods can be roughly categorized into two groups: radio tomographic imaging (RTI) and Bayesian methods. RTI, originally proposed by Wilson et al. [1], constructs a linear model between the wireless measurements and the imaging vector. The imaging vector is used to imaging the monitored region and the brightest spot in the generated image reveals the position of the target. The wireless measurements employed in RTI can be RSS variation [1], RSS variance [2,22], multi-dimensional RSS [23,24] or time-of-flight (TOF) [25,26] measurements. Since the linear model of RTI is ill-conditioned, regularization [1,27] can be utilized to solve the inverse problem. Moreover, owing to the sparse nature of the unknown vector, some work [4,13,28] employed compressive sensing (CS) method to enhance the imaging performance of RTI and reduce the number of wireless measurements at the same time.

RTI is usually combined with a Kalman filter [2,22] to track the moving targets. Bayesian DFL methods, however, directly track the target, which is accomplished by first modeling RSS measurements as the function of the target’s state and subsequently using Bayesian filter to estimate the state of the target. The current measurement models in Bayesian methods include elliptical model [14], exponential model [8,12,18], diffraction model [19,20] and three-state model [21], with complexity sorted from lowest to highest. Since the models are all nonlinear with respect to the position of the target, nonlinear filtering such as particle filtering (PF) can be applied to track the target.

Energy efficiency is a critical issue of DFL since it is closely related to the lifetime of RF sensors [29,30]. The work [31] has studied this issue, in which the author proposed to measure the RSS of the links around the target instead of all links. However, the underlying communication protocol is highly complicated since the interaction between RF sensors and BS is indispensable. Wang [26] also proposed to reduce the time and energy consumption for TOF-based DFL by only measuring the shadowed links predicted by a particle filter. In this paper, we propose a binary work mode for RF sensors, in which the RF sensors only output the link state rather than the raw wireless measurements. The binary work mode not only can remarkably reduce the overall energy consumption and also can be implemented by a quite simple protocol compared to the protocol used in [31]. In addition, we also propose the Bayesian target localization and tracking methods for the binary mode, which show satisfactory localization accuracy.

## 3. System Model

**Figure 1:**
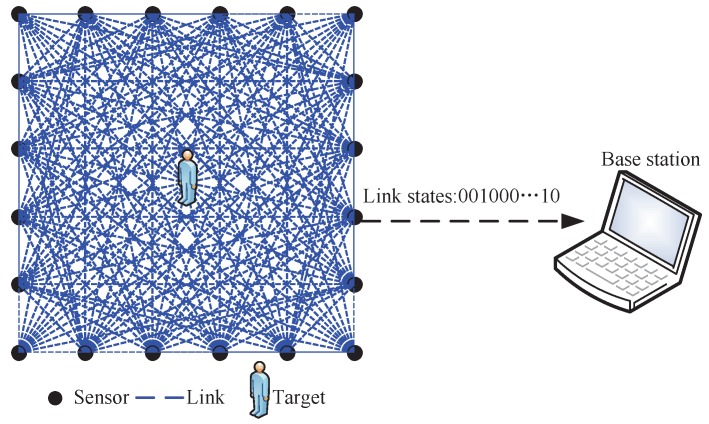
An illustration of a monitored region constituted by RF sensors.

As shown in Figure 1, *K* RF sensors are deployed at the perimeter of the monitored region and mounted on the tripods with the same height. Suppose that the position of the sensors are known in advance and the ith sensor’s position is denoted as $\alpha_{i}$, i=1,2,...,K. For simplicity, we assume that the sensors are equally spaced and the distance between two adjacent sensors on the same side is equal to *D*. RF sensors are fully connected, implying that *K* sensors can constitute $L=K\left(K-1\right)$ links. For ease of description, the links are labeled as following. Suppose that link *l* is comprised of sensor *i* and sensor *j*, where i≠j. As a result, *l* can be computed as

(1) $$l=\left\{\begin{array}{c} \left(K-1\right)\left(i-1\right)+j-1,j>i\\ \left(K-1\right)\left(i-1\right)+j,\mathrm{otherwise}. \end{array}\right.$$

**Figure 2:**
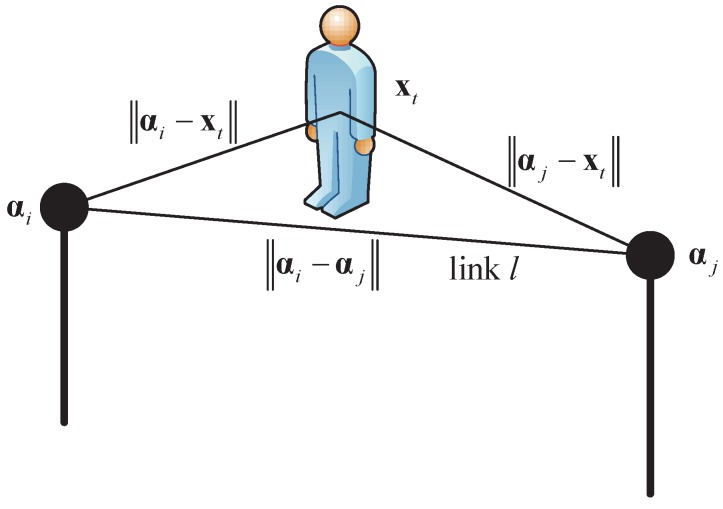
An illustration that a target shadows link *l*.

Each sensor receives the radio signals emitted by other sensor and measures the corresponding RSS. For clarity, the RSS measured before the target enters the monitored region is called static RSS and the static RSS of link *l* is denoted as ${\bar{r}}_{l}$. According to path loss model [32], ${\bar{r}}_{l}$ can be expressed as

(2) $${\bar{r}}_{l}=P_{0}\left(d_{\mathrm{Ref}}\right)-10n_{p}\log_{10}\left(\frac{d_{l}}{d_{\mathrm{Ref}}}\right),$$

where $P_{0}\left(d_{\mathrm{Ref}}\right)$ is the measured received power at a reference distance $d_{\mathrm{Ref}}$, $n_{p}$ is the path loss exponent and $d_{l}=∥\alpha_{i}-\alpha_{j}∥$ is the path length of link *l*, as shown in Figure 2.

When the target enters into the monitored region, the target will absorb part of radio signals, causing shadow fading of the link. Thus, the RSS of link *l* becomes

(3) $$r_{l,t}=P_{0}\left(d_{\mathrm{Ref}}\right)-10n_{p}\log_{10}\left(\frac{d_{l}}{d_{\mathrm{Ref}}}\right)-S_{l}\left(x_{t}\right)-v_{l,t},$$

where $x_{t}$ is the position of the target at time instant *t*, $S_{l}\left(x_{t}\right)$ is the shadowing loss due to the obstruction by the target and $v_{l,t}$ is the additive noise. The noise originates from the reflection of the target or other perturbations in the monitored region. Generally, the noise can be modeled as Gaussian distributed with zero-mean and variance $\sigma^{2}$, i.e., $v_{l,t}∼N\left(0,\sigma^{2}\right)$, where the parameter $\sigma^{2}$ can be determined depending on the actual environment.

Subtracting the static RSS in (2), we obtain the variation of RSS due to the presence of target, which can be written as

(4) $$\Delta r_{l,t}={\bar{r}}_{l}-r_{l,t}=S_{l}\left(x_{t}\right)+v_{l,t}.$$

Note that the irrelevant terms in (2) have been removed, which means that it is unnecessary to calculate these terms.

Some prior works have attempted to model the shadowing loss as a function of the target’s position, for example, elliptical model [1,13,14], exponential model [8,12] and diffraction model [7,19,33,34]. The exponential model is established through fitting extensive measurements collected from real experiments, which can be written as

(5) $$S_{l}\left(x_{t}\right)=\phi e^{-\kappa \Delta d_{l}\left(x_{t}\right)},$$

where $\Delta d_{l}\left(x_{t}\right)=∥x_{t}-\alpha_{i}∥+∥x_{t}-\alpha_{j}∥-∥\alpha_{i}-\alpha_{j}∥$ is the excess path length, ϕ is the maximum loss evaluated when $\Delta d_{l}\left(x_{t}\right)=0$ and κ is the decaying factor. The parameters ϕ and κ are usually determined in the experiment. We can see that the shadowing loss is exponentially decreasing as the growth of the excess path length. Considering that the exponential model holds both satisfactory accuracy and analytical property, the exponential model will be adopted in this paper.

In the traditional work mode of RF sensors [1], the sensors directly send raw measurements $r_{l,t}$ of all links to the BS. The traditional mode is simple but lacks energy efficiency, as will be explained in Section 3. In the binary mode, when a sensor obtains the variation of RSS $\Delta r_{l,t}$, it first compares $\Delta r_{l,t}$ with the predefined threshold γ. If $\Delta r_{l,t}$ exceeds the threshold, the link will be detected to be obstructed by the target and the sensor will send the corresponding link number to the BS, otherwise the transmitter of sensor will keep inactive. Therefore, the state of link *l* can be written as

(6) $$z_{l,t}=\left\{\begin{array}{c} 1,\mathrm{if}\;\Delta r_{l,t}\geq \gamma \\ 0,\;\;\;\mathrm{otherwise}. \end{array}\right.$$

We can see that the sensor only provides the link state which indicates whether the link is obstructed or not. Therefore, the sensor can be regarded as *binary* in this sense. The detection threshold γ can be chosen in terms of the probability of false alarm $P_{FA}$. Recall that the noise $v_{l,t}$ can be modeled as zero-mean Gaussian distributed noise with variance $\sigma_{n}^{2}$, which can be estimated by measuring the variance of RSS fluctuation when the target is moving away from a link. Given the probability of false alarm $P_{FA}$, the threshold can be computed as

(7) $$\gamma =\sigma_{n}Q^{-1}\left(P_{FA}\right)$$

where $Q\left(\cdot \right)$ denotes the Q function [35]. Alternatively, the threshold can be empirically chosen according to the environment where the experiment is conducted.

Obviously, due to the influence of the noise, the state of a link is not deterministic but random and can be characterized by probability theory. According to detection theory, conditioned on the target’s position $x_{t}$, $p\left(z_{l,t}=1|x_{t}\right)$ and $p\left(z_{l,t}=0|x_{t}\right)$ can be given by

(8) $$\begin{array}{c} p\left(z_{l,t}=1|x_{t}\right)=p\left(\Delta r_{l,t}\geq \gamma \right)\\ =\int_{\gamma }^{\infty }\frac{1}{\sqrt{2\pi }\sigma }e^{-\frac{{\left(\Delta r_{l,t}-S_{l}\left(x_{t}\right)\right)}^{2}}{2\sigma^{2}}}d\Delta r_{l,t}\\ =Q\left(\frac{\gamma -S_{l}\left(x_{t}\right)}{\sigma }\right), \end{array}$$

and

(9) $$p\left(z_{l,t}=0|x_{t}\right)=p\left(\Delta r_{l,t}<\gamma \right)=1-Q\left(\frac{\gamma -S_{l}\left(x_{t}\right)}{\sigma }\right).$$

## 4. Efficiency Analysis

In most outdoor applications, RF sensors have to be powered by batteries. Therefore, the reduction of power consumption is the key to prolonging the lifetime of RF sensors. In this section, the frame structures of the two work modes of RF sensors are described and the comparison of energy consumption between the two modes is also presented.

Figure 1 shows a typical structure of a DFL system, which mainly consists of two components: distributed RF sensors and BS. The job of distributed RF sensors is to measure the RSS of the links and broadcast the measurements. The BS station receives the measurements and forwards the measurement to a central computer via USB or serial port for post-processing. In the RF sensor network, each sensor is assigned a unique ID which controls the transmitting order of the sensors. To measure the RSS of the all links, the sensors should broadcast the packet in turns. For each sensor, it constitutes $\left(K-1\right)$ links with the remaining sensors. Therefore, each sensor needs to maintain the RSS of $\left(K-1\right)$ links.

**Figure 3:**
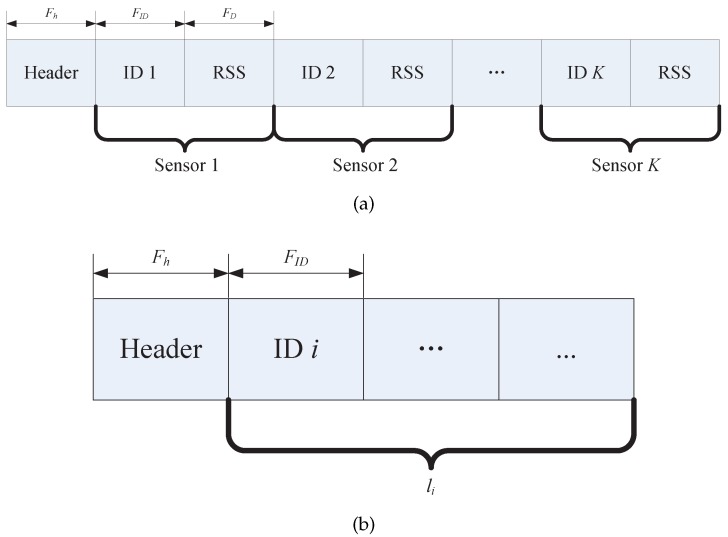
The frame structure of transmitted packet in the (**a**) traditional mode; and (**b**) binary mode.

In the traditional mode [1], the BS receives the packet at the same time when the sensor broadcasts. The traditional frame structure of transmitted packet thus can be designed as Figure 3a, which consists of three fields: header, sensor ID and RSS measurements. The header generally includes the ID of the transmitting sensor and some other necessary overhead. We can see that the frame length of the packet in the traditional mode is fixed.

However, in the binary mode, as shown in Figure 3b, the frame of transmitted packet only contains two fields: header and IDs of the sensors which provide “1” state of links. Therefore, the packet in the binary mode has variable length. The BS can distinguish the obstructed links according to the IDs of transmitting sensor in the header and the IDs in the packet.

Suppose the length of the header, ID and RSS measurement are $F_{h}$ Bytes, $F_{ID}$ Bytes and $F_{D}$ Bytes, respectively. Accordingly, the frame length in the traditional mode is $\eta_{1}=F_{h}+\left(K-1\right)\left(F_{ID}+F_{D}\right)$. Considering that the frame length of all sensors are equal, the total amount of transmitted data for completing one round of measurement in the traditional mode is

(10) $$\eta_{1}^{Total}=K\eta_{1}=K\left[F_{h}+\left(K-1\right)\left(F_{ID}+F_{D}\right)\right].$$

We can see that the total amount is approximately proportional to $K^{2}$. Returning to the binary mode, the frame length of the ith sensor is $\eta_{2}\left(i\right)=F_{h}+l_{i}F_{ID}$, where $l_{i}$ is the number of obstructed links detected by sensor *i* from its $\left(K-1\right)$ links. Therefore, the total amount of transmitted data in the binary mode is

(11) $$\eta_{2}^{Total}=\sum_{i=1}^{K}\eta_{2}\left(i\right)=\sum_{i=1}^{K}\left(F_{h}+l_{i}F_{ID}\right)=KF_{h}+M\left(x_{t}\right)F_{ID},$$

where $M\left(x_{t}\right)=\sum_{i=1}^{K}l_{i}$ is the total number of the obstructed links in the network. As we have mentioned, the link state is random due to noise. Hence, $M\left(x_{t}\right)$ can be seen as a random variable with mean of

(12) $$\bar{M\left(x_{t}\right)}=\sum_{l=1}^{L}p\left(\Delta r_{l,t}\geq \gamma \right)=\sum_{l=1}^{L}Q\left(\frac{\gamma -S_{l}\left(x_{t}\right)}{\sigma }\right).$$

**Figure 4:**
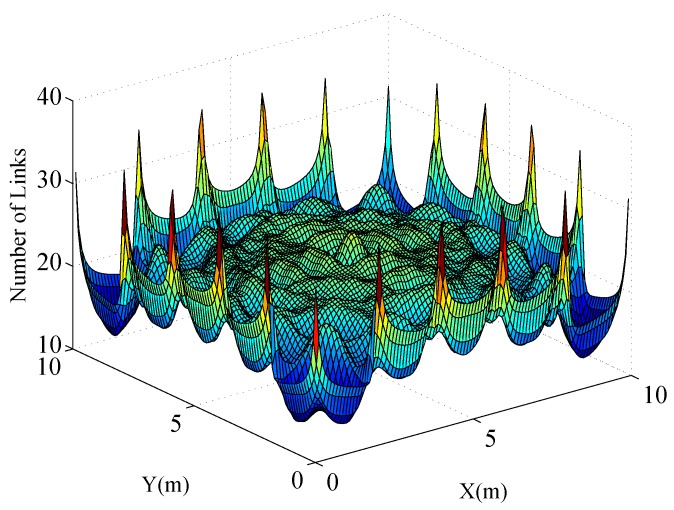
Distribution of $\bar{M\left(x_{t}\right)}$ in the monitored region.

From (12), we can see that $\bar{M\left(x_{t}\right)}$ depends on the position of the target. Figure 4 shows the value of $\bar{M\left(x_{t}\right)}$ when the target stands at different positions within the monitored region. The parameters in this simulation are chosen as K=20, ϕ=6dB, κ=20, D=2m, σ=2dB, and γ=4dB. From Figure 4 we can see that the maximum of $\bar{M\left(x_{t}\right)}$ is about 34.8 when the target is at the position of the sensors, whereas $\bar{M\left(x_{t}\right)}$ ranges from 15 to 30 when the target is at other positions. Although $\bar{M\left(x_{t}\right)}$ obtains the maximum value at the position of sensors, it still seems to be insignificant compared to the total number of links L=380.

**Figure 5:**
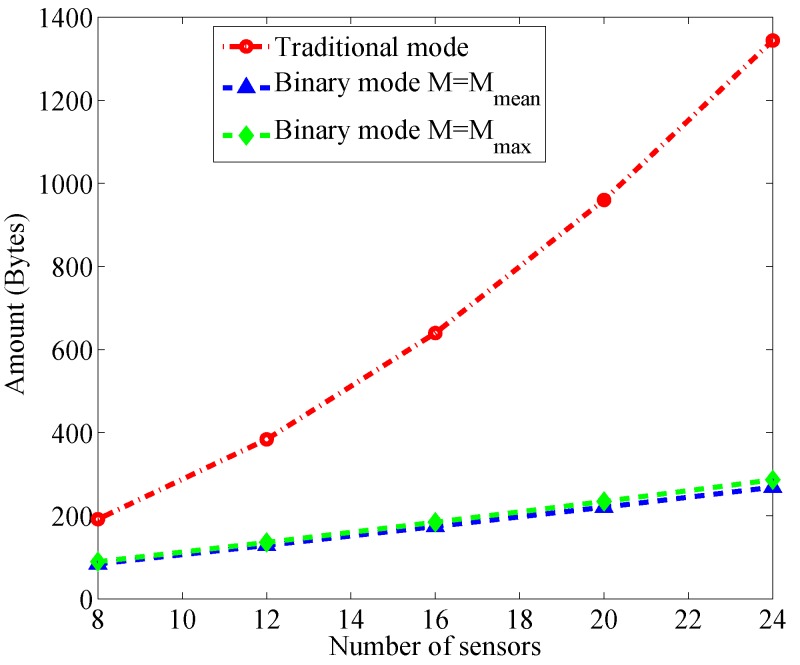
Tha amount of transmitted data for the two modes versus number of sensors.

Denote $M_{\mathrm{mean}}=E_{x_{t}}\left[\bar{M\left(x_{t}\right)}\right]$ as the average number of the obstructed links and $M_{\max}\;=\;\max_{x_{t}}\;\bar{M\left(x_{t}\right)}$ as the maximum, which can be regarded as the worst case. Figure 5 shows the total amount of data delivery in the two modes, respectively, where $F_{h}=10$ Bytes, $F_{ID}=F_{D}=1$ Byte.

We can see that $\eta_{1}^{Total}$ is approximately proportional to $K^{2}$ whereas $\eta_{2}^{Total}$ linearly increases with *K*. As the number of sensors grows, the gap between $\eta_{1}^{Total}$ and $\eta_{2}^{Total}$ becomes wider. For example, when K=40, $\eta_{1}^{Total}=3520$ Bytes whereas $\eta_{2}^{Total}=470$ Bytes when $\bar{M\left(x_{t}\right)}=M_{mean}$ and $\eta_{2}^{Total}=501$ Bytes when $\bar{M\left(x_{t}\right)}=M_{\max}$, meaning that the amount of transmitted data has been reduced remarkably even for the worst case of $\eta_{2}^{Total}$. In RF sensor network, less amount of transmitted data implies lower energy consumption if emitted power of all sensors is fixed. Therefore, the binary mode is much more energy efficient and thus more suitable for power constrained sensors.

## 5. Target Localization

The ultimate goal of deploying RF sensors is to localize the target. Unfortunately, the existing localization methods such as RTI [1] are mainly formulated based on the traditional mode and thus these methods cannot be directly applied to the binary mode. Hence, it is necessary to develop the localization method suitable for the binary work mode of sensors.

In the context of localization, two scenarios are commonly encountered. One is that the target is stationary in the monitored region and the other scenario is that the target keeps moving. The localization in the second scenario is also known as target tracking. This section will discuss the localization problem under the first scenario and the second scenario will be considered in the next section.

### Grid-Based Maximum Likelihood (GML) Localization

We stack the link states into a column vector as $z_{t}={\left[z_{1,t},z_{2,t},...,z_{l,t}\right]}^{T}$. As we know that the optimal estimation of $x_{t}$ is given by Maximum a Posterior (MAP) estimator. Since we have no prior knowledge of $x_{t}$, MAP estimation is equivalent to ML estimation, which can be written as

(13) $${\hat{x}}_{t}^{\mathrm{ML}}=\arg\max_{x_{t}}\;p\left(z_{t}|x_{t}\right).$$

If *L* links are assumed to be mutually independent, the likelihood function can also be expressed as

(14) $$p\left(z_{t}|x_{t}\right)=p\left(z_{1,t},z_{2,t},...,z_{l,t}|x_{t}\right)=\prod_{l=1}^{L}p\left(z_{l,t}|x_{t}\right).$$

From the link state model in (6), we know that $p\left(z_{l,t}|x_{t}\right)$ is a highly nonlinear function with respect to $x_{t}$. Thus, maximizing $p\left(z_{t}|x_{t}\right)$ involves nonlinear optimization. Iterative methods such as gradient descent approaches [15] have large computation complexity and also lack of global convergence. An alternative approach is to use the grid search method which divides the target state space into discrete grids and finds the grid maximizing the likelihood function. The grid based method can effectively overcome the difficulty of local convergence. However, the amount of computation exponentially increases as the growth of resolution. Fortunately, we will later prove that the most of the computation can be performed offline and thus the online computation burden is low.

**Figure 6:**
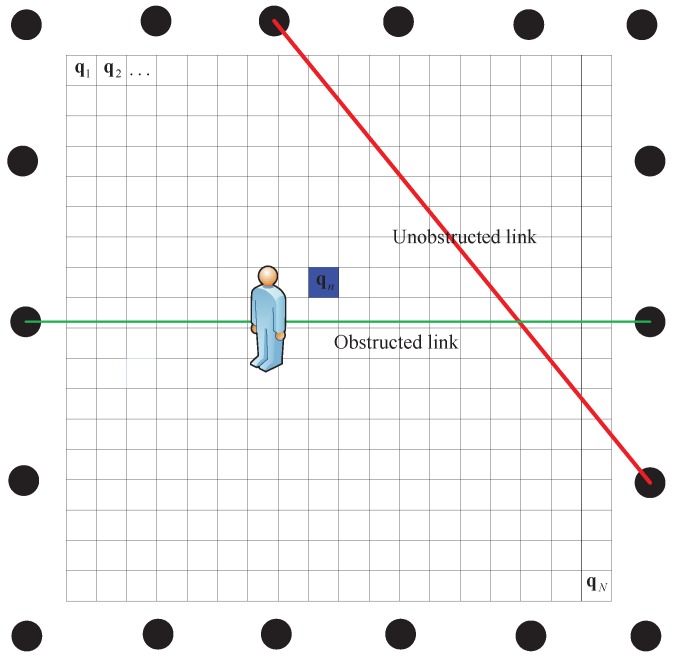
An illustration of GML.

In grid search method, the monitored region is uniformly divided into *N* grids and the size length of each grid is denoted as Δυ, as illustrated in Figure 6. The coordinate of the center of the nth grid is denoted as $q_{n}$. Thus, the GML estimation can be rewritten as

(15) $${\hat{x}}_{t}^{\mathrm{GML}}=\arg\max_{q_{n}}\;p\left(z_{t}|q_{n}\right).$$

We partition the links into two sets, namely, $\ell_{t}^{0}$ and $\ell_{t}^{1}$, where $\ell_{t}^{0}=\left\{l:z_{l,t}=0\right\}$ is the set of unobstructed links and $\ell_{t}^{1}=\left\{l:z_{l,t}=1\right\}$ is the set of obstructed links. It is easy to verify that $\ell_{t}^{0}⋃\ell_{t}^{1}=\left\{1,2,...,L\right\}$. From (8) and (9), we can obtain the conditional probabilities $p\left(z_{l,t}=0|q_{n}\right)=1-Q\left(\frac{\gamma -S_{l}\left(q_{n}\right)}{\sigma }\right)$ and $p\left(z_{l,t}=1|q_{n}\right)=Q\left(\frac{\gamma -S_{l}\left(q_{n}\right)}{\sigma }\right)$. Thus, the likelihood for the target locating at the nth grid can be calculated as

(16) $$p\left(z_{t}|q_{n}\right)=\prod_{l\in \ell_{t}^{1}}Q\left(\frac{\gamma -S_{l}\left(q_{n}\right)}{\sigma }\right)\cdot \prod_{l\in \ell_{t}^{0}}\left[1-Q\left(\frac{\gamma -S_{l}\left(q_{n}\right)}{\sigma }\right)\right].$$

To simplify computation, log-likelihood is frequently used, which can be expressed as

(17) $$\begin{array}{c} {\hat{x}}_{t}^{\mathrm{GML}}=\arg\max_{q_{n}}\;\log p\left(z_{t}|q_{n}\right)\\ =\arg\max_{q_{n}}\;\sum_{l\in \ell_{t}^{1}}\log Q\left(\frac{\gamma -S_{l}\left(q_{n}\right)}{\sigma }\right)\\ +\sum_{l\in \ell_{t}^{0}}\log\left[1-Q\left(\frac{\gamma -S_{l}\left(q_{n}\right)}{\sigma }\right)\right]. \end{array}$$

The majority of the computation consists of computing $\log Q\left(\frac{\gamma -S_{l}\left(q_{n}\right)}{\sigma }\right)$ and $\log\left[1-Q\left(\frac{\gamma -S_{l}\left(q_{n}\right)}{\sigma }\right)\right]$ for all grids of each link. Fortunately, the two terms are independent of the link state $z_{t}$, meaning that they can be computed in advance and stored in the memory. After the arrival of the link state, $\log Q\left(\frac{\gamma -S_{l}\left(q_{n}\right)}{\sigma }\right)$ and $\log\left[1-Q\left(\frac{\gamma -S_{l}\left(q_{n}\right)}{\sigma }\right)\right]$ are loaded for calculating the log-likelihood. Hence, the online computational complexity of GML is low.

The procedure of GML localization can be summarized as follows:

- Offline phase: Calculate $\log Q\left(\frac{\gamma -S_{l}\left(q_{n}\right)}{\sigma }\right)$, $\log\left[1-Q\left(\frac{\gamma -S_{l}\left(q_{n}\right)}{\sigma }\right)\right]$ for l=1,2,...,L and n=1,2,...,N, and store them into memory.
- Online phase:
  1. Partition the links into sets $\ell_{t}^{0}$ and $\ell_{t}^{1}$ in terms of their states.
  2. Compute the log-likelihood function according to (17) by loading the offline calculations.
  3. Select the grid maximizing the log-likelihood function as the ML estimation ${\hat{x}}_{t}^{\mathrm{ML}}$.

## 6. Target Tracking

In this section, we will discuss another scenario, i.e., target tracking in RF sensor network. First the motion model of the target will be presented and next the particle filter is adopted to track the target.

### 6.1. Motion Model

Denote $X_{t}={\left(x_{t},y_{t},{\dot{x}}_{t},{\dot{y}}_{t}\right)}^{T}\in {ℜ}^{4\times 1}$ is the state of the target at time instant *t*, where ${\dot{x}}_{t}$ and ${\dot{y}}_{t}$ are the velocities in *x* direction and *y* direction, respectively. $\Delta t=\frac{\eta_{2}^{Total}}{R}$ is the updating time between two consecutive rounds of measurement, where *R* is the data rate. For simplicity, we assume that the dynamic of the target can be described by constant velocity (CV) motion model [36]. Thus, the state transition equation can be written as

(18) $$X_{t}=FX_{t-1}+Bu_{t},$$

where the matrix F and matrix B are given by

(19) $$F=\left[\begin{array}{cccc} 1 & 0 & \Delta t & 0\\ 0 & 1 & 0 & \Delta t\\ 0 & 0 & 1 & 0\\ 0 & 0 & 0 & 1 \end{array}\right],B=\left[\begin{array}{cc} {\Delta t}^{2}/2 & 0\\ 0 & {\Delta t}^{2}/2\\ \Delta t & 0\\ 0 & \Delta t \end{array}\right],$$

and $u_{t}\in {ℜ}^{2\times 1}$ is the acceleration noise, which is assumed to be Gaussian distributed with zero-mean and covariance matrix $R=\mathrm{diag}\left(\sigma_{x}^{2},\sigma_{y}^{2}\right)$, where $\sigma_{x}^{2}$ and $\sigma_{y}^{2}$ are the variance of acceleration noise in *x* direction and *y* direction, respectively. For more complicated motion models, the reader can refer to [36] for more details. As the measurement $z_{t}$ is independent of the velocity of the target, the likelihood $p\left(z_{t}|X_{t}\right)$ is equal to $p\left(z_{t}|x_{t}\right)$.

### 6.2. Particle Filter Tracking

In stationary target scenario, one snapshot of link states $z_{t}$ is sufficient to localize the target. However, in target tracking scenario, current snapshot as well as previous snapshots should be jointly employed to estimate the state of the target. It is well known that the optimal state estimation of the target is given by Bayesian filter which maximizes the posterior probability, i.e.,

(20) $${\hat{X}}_{t}^{\mathrm{MAP}}=\arg\max_{X_{t}}\;p\left(X_{t}|z_{1:t}\right),$$

where $z_{1:t}=\left\{z_{1},z_{2},...,z_{t}\right\}$ are the link states up to time instant *t*. According to Bayes’ rule, $p\left(X_{t}|z_{1:t}\right)$ can be recursively calculated as

(21) $$\begin{array}{c} p\left(X_{t}|z_{1:t-1}\right)=\int p\left(X_{t}|X_{t-1}\right)p\left(X_{t-1}|z_{1:t-1}\right)dX_{t-1}\\ p\left(X_{t}|z_{1:t}\right)=\frac{p\left(z_{t}|X_{t}\right)p\left(X_{t}|z_{1:t-1}\right)}{p\left(z_{t}|z_{1:t-1}\right)}, \end{array}$$

where $p\left(z_{t}|z_{1:t-1}\right)=\int p\left(z_{t}|X_{t}\right)p\left(X_{t}|z_{1:t-1}\right)dX_{t}$. The above recursion is difficult to implement because the analytical solution is intractable. In recent years, sequential Monte Carlo (SMC) or also called PF has proven to be a powerful tool to overcome the difficulty of nonlinear filtering [16,17]. PF attempts to approximate the distribution $p\left(X_{t}|z_{1:t}\right)$ with weighted particles. Specifically, suppose that $N_{PF}$ is the number of particles, $X_{t}^{i}$ and $w_{t}^{i}$ are the ith particle and its associated weight, respectively, the posterior distribution $p\left(X_{t}|z_{1:t}\right)$ can be approximated as

(22) $$p\left(X_{t}|z_{1:t}\right)\approx \sum_{i=1}^{N_{PF}}w_{t}^{i}\delta \left(X_{t}-X_{t}^{i}\right),$$

where $\delta \left(\cdot \right)$ is Dirac Delta function.

The weight of particle is updated by

(23) $$w_{t}^{i}\propto w_{t-1}^{i}\frac{p\left(z_{t}|X_{t}^{i}\right)p\left(X_{t}^{i}|X_{t-1}^{i}\right)}{\pi \left(X_{t}^{i}|X_{t-1}^{i},z_{t}\right)},$$

where $\pi \left(X_{t}\right)$ is the proposal distribution. If we choose the proposal to be $\pi \left(X_{t}^{i}|X_{t-1}^{i},z_{t}\right)=p\left(X_{t}^{i}|X_{t-1}^{i}\right)$, weight updating can be greatly simplified as

(24) $$\begin{array}{c} w_{t}^{i}\propto w_{t-1}^{i}p\left(z_{t}|X_{t}^{i}\right)=w_{t-1}^{i}\prod_{l=1}^{L}p\left(z_{l,t}|X_{t}^{i}\right)=w_{t-1}^{i}\prod_{l=1}^{L}p\left(z_{l,t}|x_{t}^{i}\right)\\ \;=w_{t-1}^{i}\prod_{l\in \ell_{t}^{1}}Q\left(\frac{\gamma -S_{l}\left(x_{t}^{i}\right)}{\sigma }\right)\cdot \prod_{l\in \ell_{t}^{0}}\left[1-Q\left(\frac{\gamma -S_{l}\left(x_{t}^{i}\right)}{\sigma }\right)\right]. \end{array}$$

The weight should be normalized to unity, i.e.,

(25) $$w_{t}^{i}\leftarrow \frac{w_{t}^{i}}{\sum_{i=1}^{N_{PF}}w_{t}^{i}}.$$

The drawback of PF is degeneracy which only some particles have large weights and the weight of other particles are negligible, which makes the particles lack of diversity. Degeneracy will greatly degrade the performance of PF and must be avoided. A general metric to evaluate the level of degeneracy is the effective sample size $N_{eff}\approx \frac{1}{\sum_{i=1}^{N_{PF}}{\left(w_{t}^{i}\right)}^{2}}$ [16]. If $N_{eff}$ is less than a threshold $N_{th}$, degeneracy is thought to take place. The approach to deal with the particle degeneracy is resampling which replaces large weight particle with some equal weight particles and drops little weight particles.

After performing those steps, the target state estimation and its covariance are given by

(26) $$\begin{array}{c} {\hat{X}}_{t}^{\mathrm{MAP}}\approx \sum_{i=1}^{N_{PF}}w_{t}^{i}X_{t}^{i}\\ \mathrm{cov}\left(X_{t}\right)\approx \sum_{i=1}^{N_{PF}}w_{t}^{i}\left(X_{t}^{i}-{\hat{X}}_{t}\right){\left(X_{t}^{i}-{\hat{X}}_{t}\right)}^{T}. \end{array}$$

## 7. Experimental Results

We conduct real experiments to verify the effectiveness of the proposed method. The RF sensor network is comprised of CC2530 nodes, which are fully compatible with IEEE 802.15.4 standard and work at 2.4 GHz frequency band. CC2530 node is able to provide RSS measurement via its internal module. The transmitting power of CC2530 sensor is set to 4.5 dBm. The RF sensors were equipped with directional antennas [37] which have 110 degrees of horizontal beamwidth to mitigate the interference outside the monitored region.

**Figure 7:**
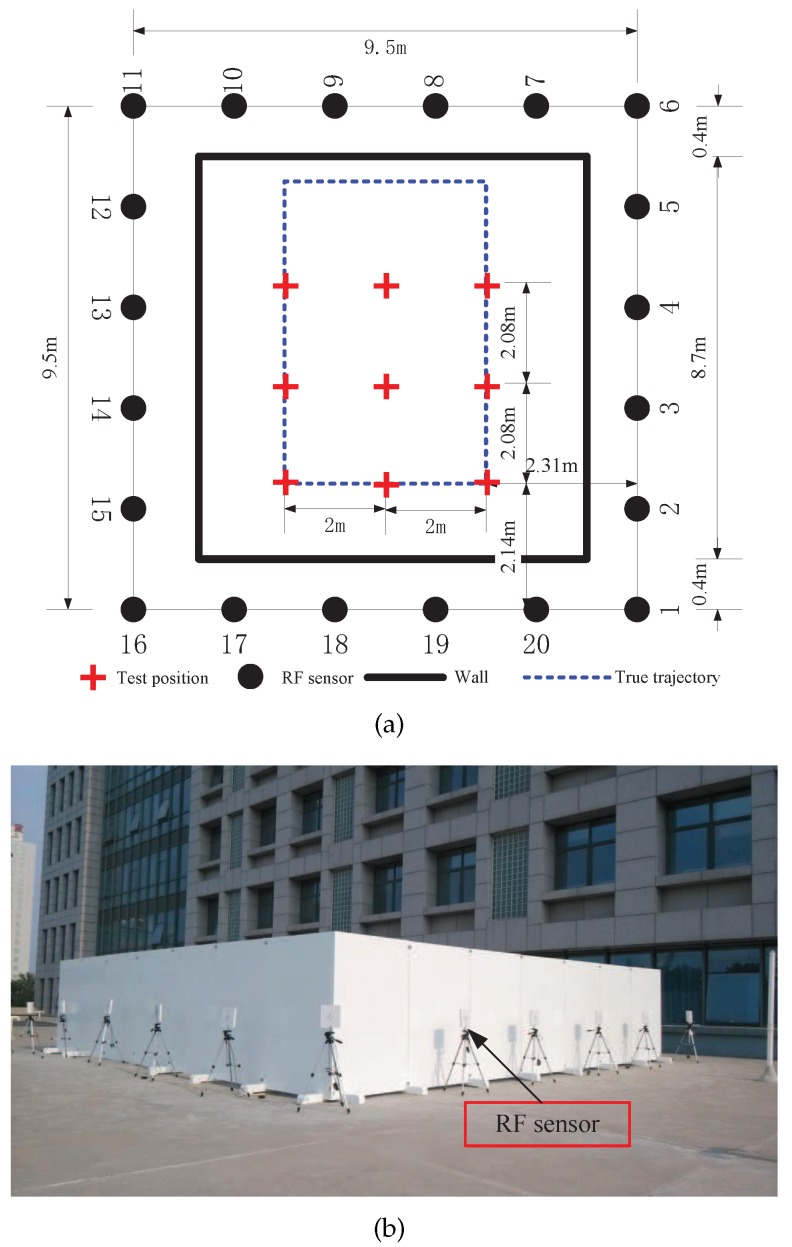
The outdoor experimental environment: (**a**) a sketch of the experimental layout and (**b**) photography of the experimental environment.

**Figure 8:**
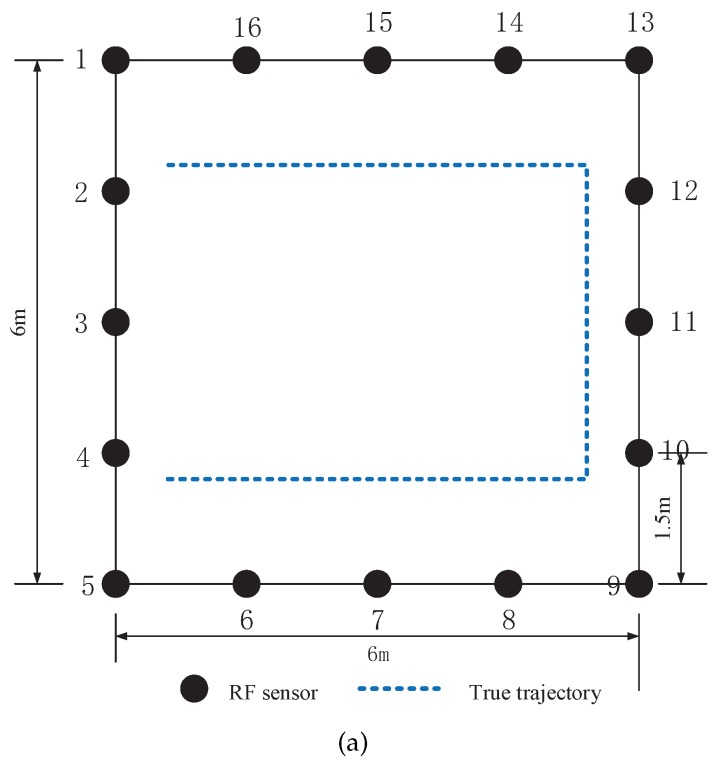

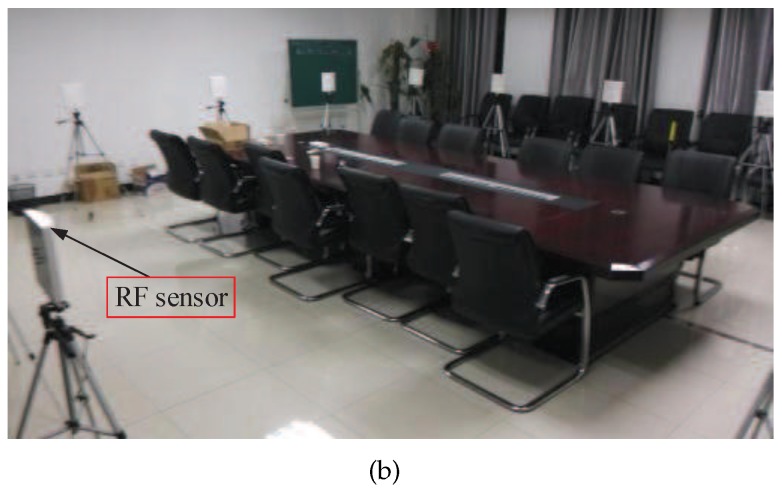
The indoor experimental environment: (**a**) a sketch of the experimental layout and (**b**) photography of the experimental environment.

The experiments are carried out in two different environments. The first environment is an outdoor environment, as shown in Figure 7. 20 RF sensors were uniformly placed at the perimeter of the monitored region. The distance between two adjacent sensors was D=1.9 m. Hence, the area of the square monitored region was 9.5 m × 9.5 m = 90.25 $m^{2}$. The target moved in the area surrounded by wood boards with a thickness of 3 cm and a height of 1.8 m. The second environment is a typical indoor environment, as shown in Figure 8. We can see that there were tables, chairs and walls in the environment and hence it is a multipath rich environment. 16 RF sensors constitute a monitored region of 6 m × 6 m.

### 7.1. Results of Outdoor Experiment

In the following, we will evaluate the energy efficiency and localization performance of the proposed method in the first environment.

#### 7.1.1. Efficiency Comparison

First, to compare the energy efficiency between the traditional mode and the binary mode, the sensors were allowed to work in the two modes successively. Considering that the energy consumed by all sensors during one round of measurement is proportional to the updating time because the emitted power of all sensors are equal, less updating time means lower energy consumption. Thus, updating time of a link in the traditional work mode and in the binary mode are compared. The fields length of the two types of frames are chosen as $F_{h}=17$ Bytes, $F_{ID}=1$ Byte and $F_{D}=1$ Byte. Actually, the minimum length of header is limited by IEEE 802.15.4 protocol. If users able to develop protocols regardless of IEEE 802.14.5 protocol, the header length can be remarkably reduced. Moreover, note that the link state updating time of the proposed mode is time-varying depending on the position of target. Thus, we let the target randomly move within the monitored area and the sensors measures the RSS or the states of the links simultaneously.

**Figure 9:**
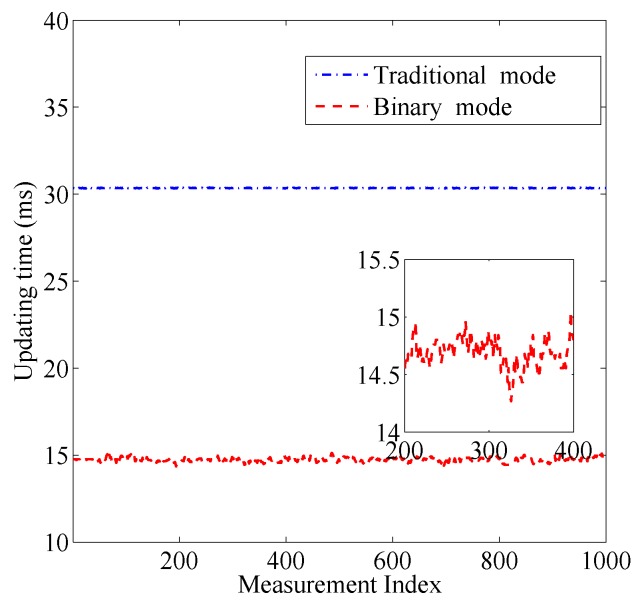
Updating time in the traditional mode and the binary mode.

Figure 9 shows the updating time in the traditional work mode and the proposed work mode during 1000 rounds of measurements, respectively. The threshold γ is set to 4 dB. We can see that the updating time in the traditional mode almost keeps unchanged to be 30.4 ms. In contrast, the updating time in the proposed work mode is only about 14.7 ms, which is reduced by 52% compared to that in the traditional work mode. Moreover, if we zoom the figure, we can see that the updating time in the proposed work mode varies when the target is moving within the monitored area because the number of obstructed links is different when the target is located at different positions.

**Figure 10:**
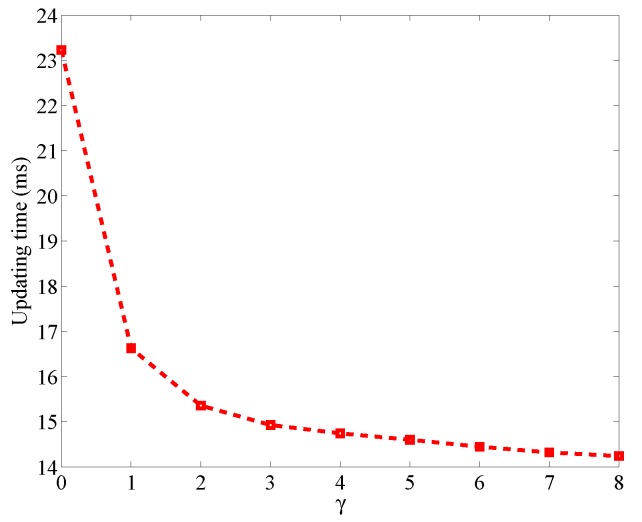
Updating time versus threshold in the binary mode.

If we increase the threshold γ, the energy efficiency can be further enhanced. Figure 10 shows the updating time of a link in the proposed work mode when the threshold ranges from 0 dB to 8 dB. We can see that the updating time can be reduced to 14 ms when the threshold is 8 dB. However, larger threshold means less links detected to be obstructed by the RF sensors, which will degrade the localization performance. Therefore, we should make a trade-off between energy efficiency and localization performance. Considering that the attenuation of RSS due to obstruction usually ranges from 5 dB to 10 dB [1], we choose the threshold as 4 dB to keep most obstructed links.

#### 7.1.2. Localization Results

First we verify the localization performance of GML estimation in single snapshot scenario. The binary work mode is applied to all the RF sensors to enhance energy efficiency. In this experiment, the updating time Δt is about 14.7 ms as shown in Figure 9. We select $N_{T}=9$ test positions in the monitored region, which are marked by red crosses, as shown in Figure 7. A person stands still at each position for a while which allows the RF sensors to measure the link states.

Localization error is a commonly employed metric to quantify the localization performance. Let $x_{t}$ denote the true position of the target at time instant *t* and ${\hat{x}}_{t}$ denote the estimation given by GML or PF. Thus, Localization error $e_{t}$ can be calculated as $e_{t}=∥x_{t}-{\hat{x}}_{t}∥$. If there are $N_{T}$ time instants of measurements, the root mean square estimation (RMSE) of position, which is written as $\mathrm{RMSE}=\sqrt{\frac{1}{N_{T}}\sum_{t=1}^{N_{T}}{∥x_{t}-{\hat{x}}_{t}∥}^{2}}$ can be used to evaluate the localization performance.

**Figure 11:**
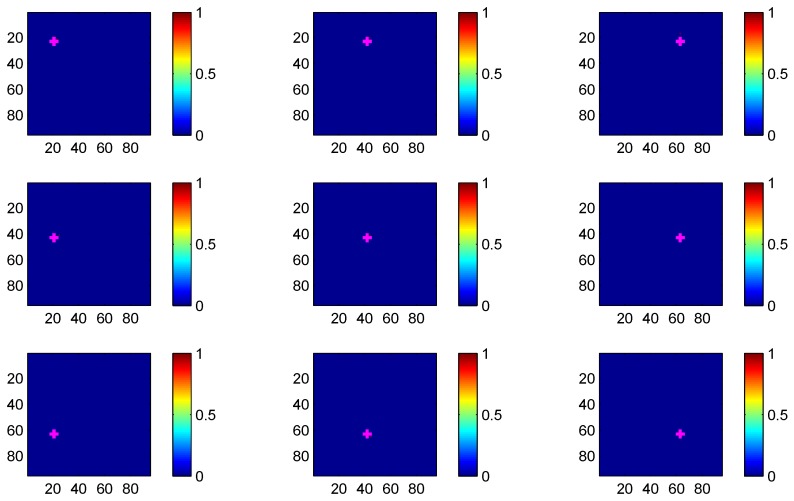
The heatmap of likelihood value for GML at the 9 test positions.

The parameters are set to ϕ=6dB, κ=20, σ=2dB and γ=4dB. The parameters of GML are chosen as Δυ=0.1m. Thus, the monitored region can be divided into 95 × 95 grids. Figure 11 shows the likelihood value $p\left(z_{t}|q_{n}\right)$ at each grid for the 9 test position, respectively. For visualization, likelihood value has been quantized into the range [0,1]. The pixel with brighter color implies larger likelihood value and the brightest pixel reveals the position of the target. In Figure 11, the true position of target is marked by cross. We can see that the brightest pixel is very close to the true position at each test position, meaning that GML achieves good localization performance. For comparison, the localization results of RTI in [1] using raw measurements are also presented. The localization results of RTI and the proposed GML method are listed in Table 1 and Table 2, respectively. We can see that the RMSE of the proposed method is 0.317 m, which merely increases 0.035 m compared to the RMSE of RTI. It means that the binary work mode does not sacrifice large localization performance.

#### 7.1.3. Tracking Results

**Figure 12:**
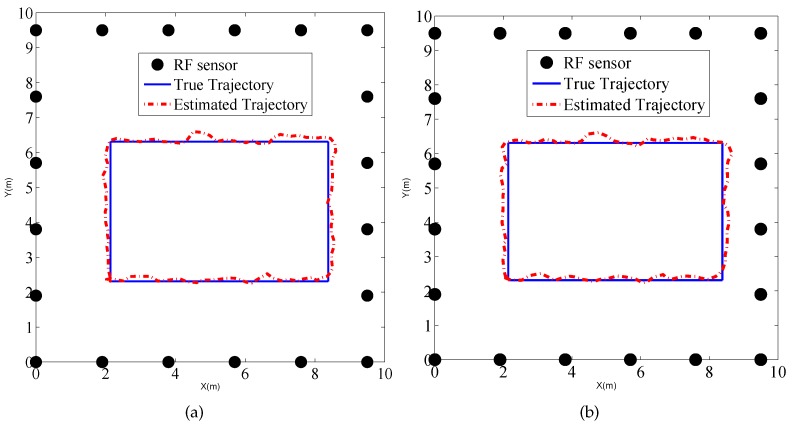
Tracking result of (**a**) traditional mode using raw measurements; and (**b**) binary mode using link states in the outdoor environment.

To explore the performance of the tracking method in the binary work mode, a person moves along the rectangular trajectory, as shown in Figure 12 at a speed of 0.5 m/s. Meanwhile RF sensors measure the states of the links and send the measurements to the local PC which runs the MATLAB routines of PF tracking algorithm.

The noise variance σ and threshold γ are still chosen as σ=2dB and γ=4dB. The motion parameters of the target are chosen as $\sigma_{x}=\sigma_{y}=0.5\;m/s^{2}$. The parameters of PF are $N_{PF}=1000$ and $N_{th}=2/3$.

**Figure 13:**
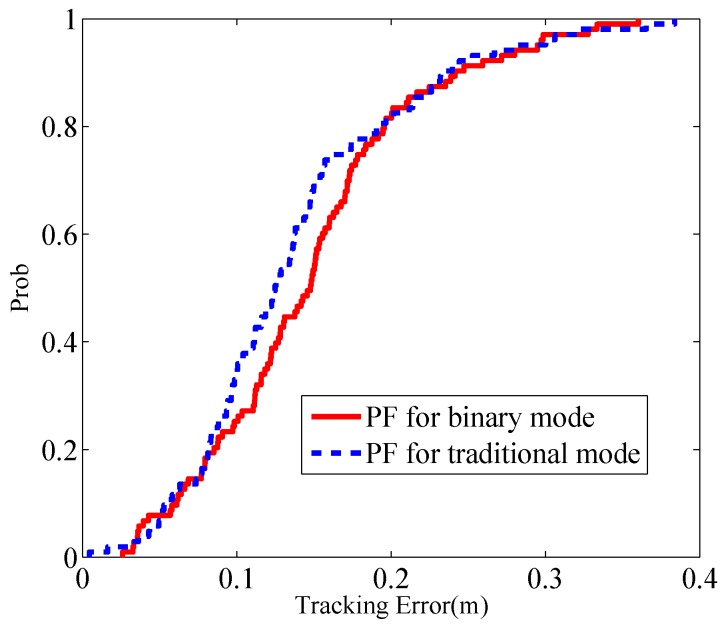
CDF of localization error.

We compare the the tracking performance using PF in the binary mode and the traditional mode [8] respectively. Figure 13 shows the cumulative distribution function (CDF) of localization error given in the two modes. The RMSE of tracking results of the binary mode and traditional mode are 0.17 m and 0.16 m, respectively. The tracking performance between the two modes seems to be insignificant, which again verifies that the binary states are sufficient to track the target without performance loss.

### 7.2. Results of Indoor Experiment

In the following, we will evaluate the energy efficiency and localization performance of the proposed method in the indoor environment.

#### 7.2.1. Efficiency Comparison

**Figure 14:**
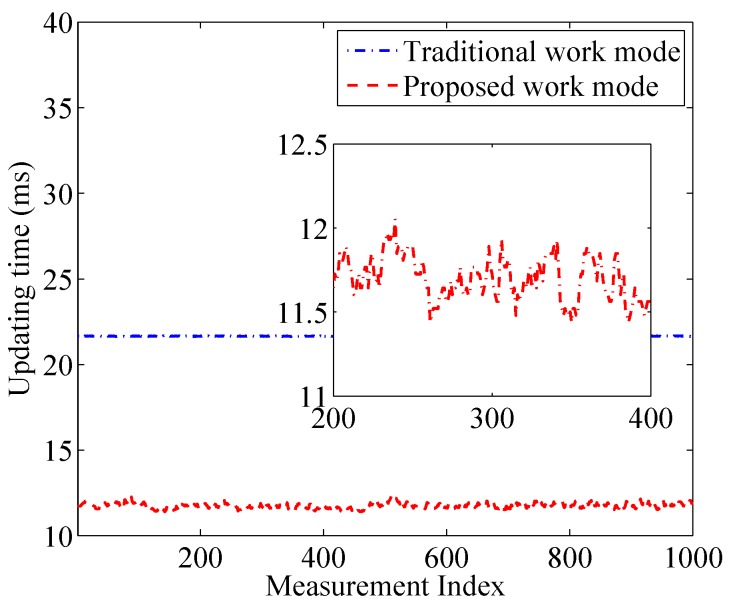
Updating time in the traditional mode and the binary mode.

Figure 14 displays the updating time in the proposed work mode and traditional work mode in the indoor environment. The threshold parameter is set to 4 dB and the other parameters are the same with those in the outdoor environment. It is shown that the updating time in the proposed work mode significantly reduces compared to that in the traditional work mode. To be more specific, the updating time in the traditional work mode is 21.65 ms and the mean updating time in our proposed work mode is 11.74 ms, decreased by 45.8%. The percentage of time reduction shrinks compared to the outdoor environment. It is because multipath fading is severe in the indoor environment, which results in more false alarms.

**Figure 15:**
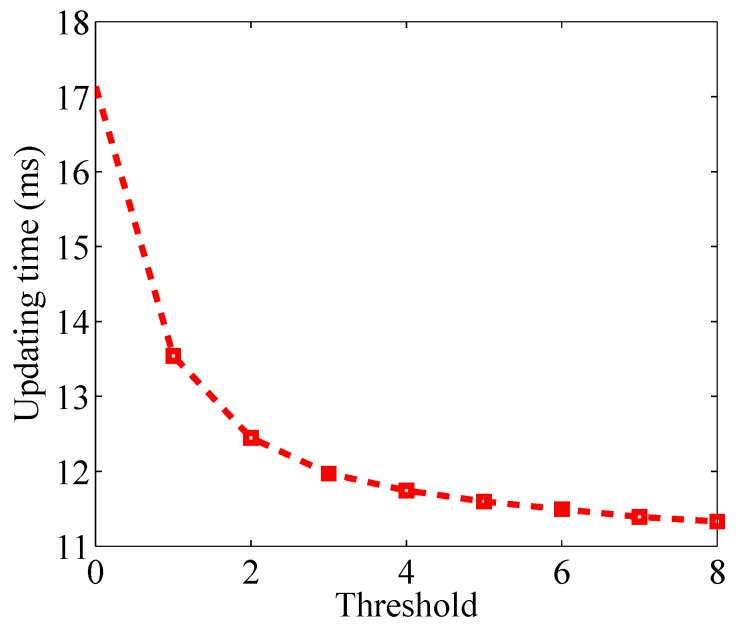
Updating time versus threshold in the binary mode.

Figure 15 shows the change of updating time when the threshold varies from 0 dB and 8 dB. We can see that, as threshold increases, the energy efficiency reduces, which is consistent with the result of outdoor environment.

#### 7.2.2. Localization and Tracking Results

**Figure 16:**
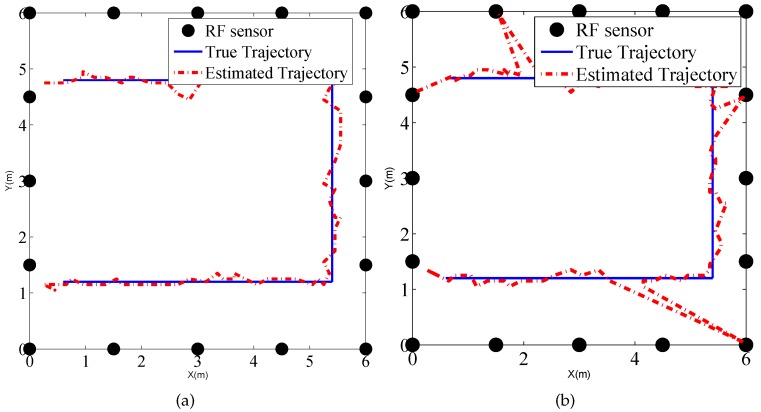
The localization result of (**a**) RTI; and (**b**) GML method.

In the second environment, the target walks along the predefined trajectory at a speed of 0.3 m/s, as shown in Figure 8. To investigate the performance of GML in the second environment, we utilize GML method to localize the target independently at each moment. Figure 16 shows the corresponding localization results of GML. For sake of comparison, we also present the localization results of RTI method which works in the traditional work mode. We can see that at most time moments, the position estimation provided by GML well agrees with the true positions of the target. At serval positions, the localization error is larger than 1 m. It occurs because multipath interference is dramatic at these positions. The RMSE of GML in the indoor environment is about 0.414 m, degraded by 23% compared to the outdoor environment.

**Figure 17:**
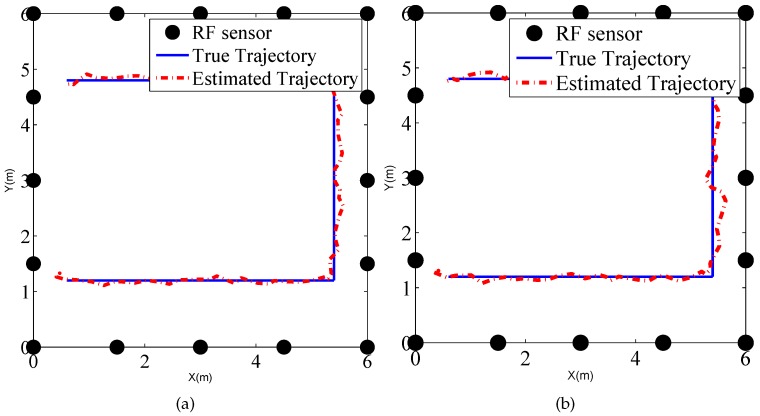
Tracking result of (**a**) traditional mode using raw measurements; and (**b**) binary mode using link states in the outdoor environment.

**Figure 18:**
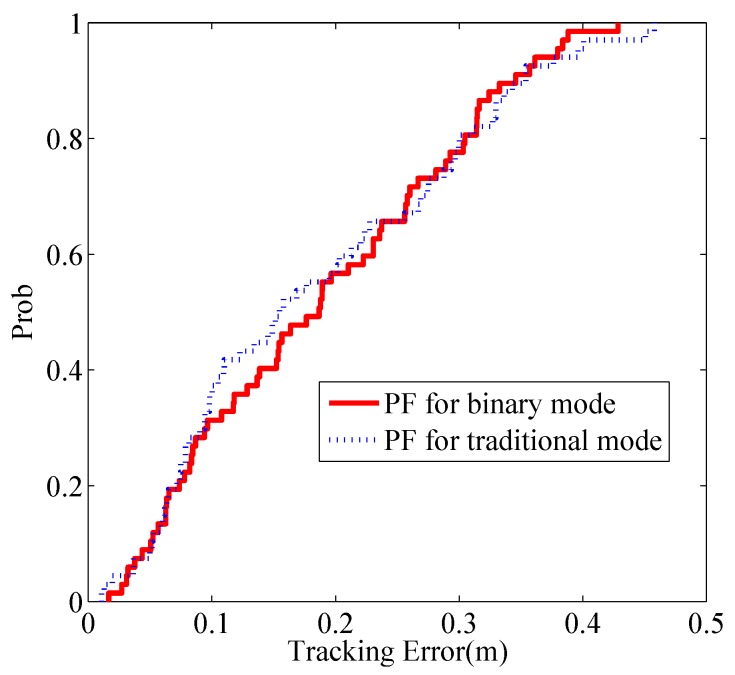
CDF of localization error.

We also present the tracking results using PF in the indoor environment, as shown in Figure 17, which gives the estimated trajectories using PF in the traditional work mode and proposed binary work mode, respectively. Moreover, the CDFs of tracking for the two methods are drawn in Figure 18. We can see that there is no significant difference between the two estimated trajectories, which implies that the tracking performance using binary work mode results in little performance degradation. The RMSE of the two methods are given by 0.21 m and 0.22 m. Therefore, it proves that the proposed binary work mode and target localization methods are also effective in the indoor environments.

## 8. Conclusions

In this paper, we first propose a binary mode of RF sensors to deal with the data transmission disaster encountered when many RF sensors are deployed in the monitored region. In the proposed binary work mode, the RF sensor only provides the link state, i.e., whether the link is obstructed or not instead of directly sending raw RSS measurements. The amount of transmitted data linearly increases with the growth of number of RF sensors, which is favorable for the batteries powered sensors. Moreover, we cope with two scenarios of target localization in the new binary mode. The first scenario is that the target is assumed to be stationary. We propose GML localization method which has low online computation burden. The aim of localization in the second scenario is to track the target. Due to the high nonlinear feature of measurement model, particle filter is employed to give the Bayesian estimation of the target’s position. Real experiments in both outdoor and indoor environment are conducted to verify the effectiveness of the proposed work mode and localization methods.

## Figures and Tables

**Table 1:** Localization Results of RTI.

Position Index	True	Estimated	Localization Error (m)
1	(2.14,2.31)	(2.40,2.05)	0.3677
2	(4.22,2.31)	(4.28,2.08)	0.2353
3	(6.30,2.31)	(6.35,1.85)	0.4627
4	(2.14,4.31)	(2.45,4.05)	0.4046
5	(4.22,4.31)	(4.25,4.15)	0.1628
6	(6.30,4.31)	(6.25,4.25)	0.0781
7	(2.14,6.31)	(2.45,6.25)	0.3158
8	(4.22,6.31)	(4.15,6.25)	0.0922
9	(6.30,6.31)	(6.35,6.25)	0.0781
		RMSE	0.2819

**Table 2:** Localization Results of GML.

Position Index	True	Estimated	Localization Error (m)
1	(2.14,2.31)	(2.3500,2.0500)	0.3342
2	(4.22,2.31)	(4.35,1.95)	0.3828
3	(6.30,2.31)	(6.35,1.85)	0.4627
4	(2.14,4.31)	(2.45,4.15)	0.3489
5	(4.22,4.31)	(4.35,4.05)	0.2907
6	(6.30,4.31)	(6.25,4.25)	0.0781
7	(2.14,6.31)	(2.45,6.25)	0.3158
8	(4.22,6.31)	(4.45,6.45)	0.2693
9	(6.30,6.31)	(6.45,6.15)	0.2193
		RMSE	0.3171
